# Newcastle disease virus vector-based SARS-CoV-2 vaccine candidate AVX/COVID-12 activates T cells and is recognized by antibodies from COVID-19 patients and vaccinated individuals

**DOI:** 10.3389/fimmu.2024.1394114

**Published:** 2024-05-30

**Authors:** Alejandro Torres-Flores, Luis Alberto Ontiveros-Padilla, Ruth Lizzeth Madera-Sandoval, Araceli Tepale-Segura, Julián Gajón-Martínez, Tania Rivera-Hernández, Eduardo Antonio Ferat-Osorio, Arturo Cérbulo-Vázquez, Lourdes Andrea Arriaga-Pizano, Laura Bonifaz, Georgina Paz-De la Rosa, Oscar Rojas-Martínez, Alejandro Suárez-Martínez, Gustavo Peralta-Sánchez, David Sarfati-Mizrahi, Weina Sun, Héctor Elías Chagoya-Cortés, Peter Palese, Florian Krammer, Adolfo García-Sastre, Bernardo Lozano-Dubernard, Constantino López-Macías

**Affiliations:** ^1^ UMAE Hospital de Especialidades, Centro Médico Nacional Siglo XXI, Instituto Mexicano del Seguro Social (IMSS), Unidad de Investigación Médica en Inmunoquímica, Ciudad de México, Mexico; ^2^ Posgrado en Inmunología, Escuela Nacional de Ciencias Biológicas, Instituto Politécnico Nacional, Ciudad de México, Mexico; ^3^ Division of Pharmacoengineering and Molecular Pharmaceutics, Eshelman School of Pharmacy, University of North Carolina at Chapel Hill, Chapel Hill, NC, United States; ^4^ Departamento de Biología Molecular y Validación de Técnicas, Instituto de Diagnóstico y Referencia Epidemiológicos (InDRE) “Dr, Manuel Martínez Báez”, Secretaría de Salud, Ciudad de México, Mexico; ^5^ Investigadores por México, Consejo Nacional de Humanidades, Ciencias y Tecnologías (CONAHCYT), Ciudad de México, Mexico; ^6^ División de Investigación en Salud, UMAE Hospital de Especialidades, Centro Médico Nacional Siglo XXI, IMSS, Cuauhtémoc, Ciudad de México, Mexico; ^7^ Servicio de Medicina Genómica. Hospital General de México “Dr. Eduardo Liceaga”, Ciudad de México, Mexico; ^8^ Coordinación de Investigación en Salud, Centro Médico Nacional Siglo XXI, IMSS, Ciudad de México, Mexico; ^9^ Laboratorio Avi-Mex S.A. de C.V., Ciudad de México, Mexico; ^10^ Department of Microbiology, Icahn School of Medicine at Mount Sinai, New York, NY, United States; ^11^ Consultora Mextrategy, S.A.S. de C.V., Ciudad de México, Mexico; ^12^ Center for Vaccine Research and Pandemic Preparedness (C-VaRPP), Icahn School of Medicine at Mount Sinai, New York, NY, United States; ^13^ Department of Pathology, Molecular and Cell-Based Medicine, Icahn School of Medicine at Mount Sinai, New York, NY, United States; ^14^ Department of Medicine, Division of Infectious Diseases, Icahn School of Medicine at Mount Sinai, New York, NY, United States; ^15^ Global Health and Emerging Pathogens Institute, Icahn School of Medicine at Mount Sinai, New York, NY, United States; ^16^ The Tisch Cancer Institute, Icahn School of Medicine at Mount Sinai, New York, NY, United States; ^17^ The Icahn Genomics Institute, Icahn School of Medicine at Mount Sinai, New York, NY, United States

**Keywords:** COVID19, antibody responses, antigenicity, T cell responses, vaccines, Newcastle Disease Virus

## Abstract

**Introduction:**

Several effective vaccines for severe acute respiratory syndrome coronavirus 2 (SARS-CoV-2) have been developed and implemented in the population. However, the current production capacity falls short of meeting global demand. Therefore, it is crucial to further develop novel vaccine platforms that can bridge the distribution gap. AVX/COVID-12 is a vector-based vaccine that utilizes the Newcastle Disease virus (NDV) to present the SARS-CoV-2 spike protein to the immune system.

**Methods:**

This study aims to analyze the antigenicity of the vaccine candidate by examining antibody binding and T-cell activation in individuals infected with SARS-CoV-2 or variants of concern (VOCs), as well as in healthy volunteers who received coronavirus disease 2019 (COVID-19) vaccinations.

**Results:**

Our findings indicate that the vaccine effectively binds antibodies and activates T-cells in individuals who received 2 or 3 doses of BNT162b2 or AZ/ChAdOx-1-S vaccines. Furthermore, the stimulation of T-cells from patients and vaccine recipients with AVX/COVID-12 resulted in their proliferation and secretion of interferon-gamma (IFN-γ) in both CD4+ and CD8+ T-cells.

**Discussion:**

The AVX/COVID-12 vectored vaccine candidate demonstrates the ability to stimulate robust cellular responses and is recognized by antibodies primed by the spike protein present in SARS-CoV-2 viruses that infected patients, as well as in the mRNA BNT162b2 and AZ/ChAdOx-1-S vaccines. These results support the inclusion of the AVX/COVID-12 vaccine as a booster in vaccination programs aimed at addressing COVID-19 caused by SARS-CoV-2 and its VOCs.

## Introduction

Severe acute respiratory syndrome coronavirus 2 (SARS-CoV-2) and its variants of concern have caused 775 million infections worldwide since 2020 (1, 2). Despite the incredible speed of coronavirus disease 2019 (COVID-19) vaccine development since 2020, there have been more than 7 million deaths from COVID-19, with an estimated excess mortality of 28.4 million deaths (1, 2). The unequal distribution of vaccines globally, especially impacting low- and middle-income countries (LMICs), underscores the urgency for further advancements in new vaccine platforms to address the disparity in vaccine access (3).

The naturally attenuated lentogenic Newcastle disease virus (NDV) LaSota strain is an avian virus with limited host range, making it incapable of causing productive multicycle infection in non-avian species, such as humans (4). Preclinical studies have assessed NDV-vectored vaccines against different coronaviruses, demonstrating their safety and ability to induce immune responses in both natural poultry hosts and non-natural mammalian challenge models (5–7).

The AVX/COVID-12 vaccine candidate is an NDV-LaSota vector-based vaccine that expresses the stabilized form of the SARS-CoV-2 spike protein. The stabilization is achieved by introducing six prolines (HexaPro-S), ensuring the protein remains in its closed conformation (8). This vaccine has demonstrated safety, immunogenicity, and protective efficacy in preclinical models (9, 10), and has the advantage of being produced in embryonated egg industrial facilities similarly to influenza vaccine production which is cheap and widely available (11). In a phase I clinical trial, the AVX/COVID-12 vaccine demonstrated safety, good tolerability, and immunogenicity in volunteers (12), supporting further clinical development of the vaccine. In a follow-up of the volunteers participating in a phase I trial, individuals received a booster dose of AVX/COVID-12 one year after the first immunization.

During the period before the AVX/COVID-12 booster, some individuals received a dose of any of the emergency-use-approved vaccines in Mexico. After being boosted with AVX/COVID-12, we observed a robust induction of neutralizing antibody responses in these subjects, suggesting that this vaccine could be used as a booster. Providing regular booster doses is vital to prevent future waves of COVID-19 and protect vulnerable groups such as the elderly, immunocompromised individuals, and those with underlying health conditions. Therefore, addressing the ongoing global vaccine supply shortage is a pressing matter that requires immediate attention (3).

However, high rates of infections in the population and the availability of approved vaccines in Mexico made the development of a phase II placebo-controlled clinical trial unethical. To provide evidence supporting the testing of this vaccine as a booster dose, we analyzed the capacity of the AVX/COVID-12 vaccine to bind antibodies and stimulate T-cell responses from individuals previously infected or vaccinated with approved vaccines.

## Materials and methods

### Study groups

The study groups consisted of adults aged 18 years or older, of both genders. Samples were collected and categorized into five groups:

1) Patients with COVID-19 in the acute phase of the disease (7 days after admission to the intensive care unit (ICU); n = 12) hospitalized at the UMAE Hospital de Especialidades, Centro Médico Nacional Siglo XXI, IMSS, before vaccines were available. 2) A group of 10 patients infected with the Omicron variant (Omicron peak, December 2021) who reported no previous infection or vaccination, from the Centro de Atención Temporal COVID-19 Tlatelolco y Morelos. For group 1 and 2 patients may have presented comorbidities such as type 2 diabetes mellitus, systemic arterial hypertension, chronic obstructive pulmonary disease, and chronic kidney disease. Other comorbidities, including cancer, immunodeficiencies, autoimmune diseases, hepatitis B and C, and/or HIV infections, as well as pregnancy, were exclusion criteria. Upon admission, all patients showing signs suggestive of COVID‐19 who consented to participate after signing an informed consent were recruited using a non-probabilistic convenience sampling method.

3) A group of individuals who had recovered from critical COVID-19 infection during the first peak of the pandemic (June-December 2020), six months after hospital discharge. 4) A group comprised of healthy volunteers vaccinated with two doses of Pfizer-BioNTech BNT162b2 (4 to 8 months after the second dose; n=15). 5) A group of volunteers who received two doses of Pfizer-BioNTech BNT162b2 boosted with AstraZeneca AZ/ChAdOx-1-S (4 to 8 months after the second dose; n=13).

Inclusion criteria for the groups 3–5 included a negative PCR test for SARS-CoV-2 with no clinical history of infection with the SARS-CoV-2 and a negative anti-N antibody test measured by ELISA. Exclusion criteria included cancer, immunodeficiencies, autoimmune diseases, hepatitis B and C, HIV infections, and pregnancy.

The sample size calculation was performed for an independent group trial, considering previously reported phase I and II clinical trials of BNT162b2 (13, 14) and AZ/ChAdOx-1-S vaccines (15) for an analysis of geometric means of antibody presence against the S protein and intracellular cytokine production in T lymphocytes after stimulation. A significance level (alpha) of 0.05 and a statistical power of 80% were established, resulting in the selection of 6 volunteers for each group initially.

### Serum collection

Venous blood samples were obtained from participants, and these samples were collected using two ethylenediaminetetraacetic acid (EDTA) tubes and one red tube (BD Vacutainer tubes, Franklin Lakes, NJ, USA) following standard phlebotomy procedures. After collection, all blood samples and their derivatives were processed in a biosafety level (BSL)-2 laboratory, with the use of appropriate personal protective equipment and safety precautions. Serum isolation involved centrifuging venous blood (collected in red tubes) at 2,000 x *g* for 10 minutes to separate the serum. The resulting serum was carefully extracted from the upper portion of the tube, aliquoted, and subsequently stored at -20°C until needed.

### Peripheral blood mononuclear cell isolation

PBMCs were isolated from venous blood collected in EDTA tubes (BD vacutainer tubes, Franklin Lakes, NJ, USA). Within 4 hours (h) of collection, PBMC isolation was conducted by density-gradient sedimentation of whole blood diluted at a 1:2 ratio in phosphate-buffered saline (PBS) at room-temperature. The diluted blood was layered over an appropriate volume of room-temperature Lymphoprep (Serumwerk Bernburg AG, DEU; cat. 07851). Then, the PBMCs were recovered, cryopreserved in a medium consisting of 10% dimethyl sulfoxide (DMSO; Sigma Aldrich, St. Louis, MO, USA) and 90% heat-inactivated fetal bovine serum (FBS; GIBCO, California, USA; cat. 11560636) and stored at -80°C until use.

### Vaccine manufacturing

The AVX/COVID-12 vaccine was cultivated in 10-day-old specific pathogen-free (SPF) chicken embryos through inoculation into the allantoic cavity, using 10^3.3^ 50% egg infectious dose (EID_50_)/0.1 mL of the production seed. The embryos were incubated for 72 h at 37°C with 60–70% humidity. After incubation, the embryos were refrigerated for a minimum of 12 h, and the allantoic fluid (AF) was then aseptically harvested. The AF underwent clarification through filters, was concentrated by a factor of 10X using 300 kDa cassettes and was subsequently diluted in 20 volumes of PBS. The resulting AF was frozen and stored at −70°C until needed. The experimental AVX/COVID-12 vaccine, along with NDV-V (empty vector), was provided frozen in 2 mL vials. The vaccine was produced in Avimex’s good manufacturing practice facilities in Mexico City.

### Antibody analysis by enzyme-linked immunosorbent assay

The analysis of specific anti-spike IgG antibodies was conducted using ELISA. Each plate was coated with NDV-V, AVX/COVID-12, or the ancestral receptor binding domain (RBD) of SARS-CoV-2 at a concentration of 10 µg/mL. Serum samples, prediluted at 1:40, were transferred to the plate (200 µL), and then 1:2 serial dilutions were performed. The plates were incubated at 37°C for 60 minutes and subsequently washed with tris-buffered saline (TBS)-Tween 20 buffer. A 1:4000 dilution of anti-human IgG coupled to horseradish peroxidase (HRP; MyBioSource’s, San Diego, USA; cat. MBS440121) was added to the plates, followed by an incubation at 37°C for 60 minutes. To stop the reaction, 2.5 N sulfuric acid was added, and the optical density (OD) was read at 450 nm using the Epoch Microplate Spectrophotometer (BioTek Laboratories, Seattle, Washington, USA) within 10 minutes after adding the stop solution. The antibody titer was determined as three times the value of the negative controls, and titers were represented as -Log2x40.

### T-cell proliferation assay

PBMCs were washed twice with 1X PBS to eliminate the cryopreservation medium, and the cell pellet was resuspended and adjusted to 5 x 10^6^ cells/mL. Subsequently, proliferation staining was carried out using the CellTrace Violet reagent (Invitrogen, Massachusetts, USA; cat. C34557) at 37°C for 20 min. Every 5 min, the cells were vortexed to ensure uniform staining. Following staining, the cells were washed with 1X PBS and then with Roswell Park Memorial Institute (RPMI) 1640 culture medium (GIBCO, Thermo Fisher, Massachusetts, USA) without supplementation. After this incubation period, the stained cells were harvested, washed with fresh medium, counted, and cultivated in 96-well plates (5 x 10^5^ cells/well). Subsequently, they were stimulated separately with 10 µg of NDV-V, AVX/COVID-12 or 30 nM of a mixture of 10–15 amino acid long peptides derived from the SARS-CoV-2 spike protein (PepTivator, Miltenyi Biotec, North Rhine-Westphalia, Germany). In parallel, stained and unstimulated cells were cultivated at the same concentration as the controls. After this step, the cells were cultured in RPMI 1640 with 10% FBS and incubated for 72 h at 37°C. The cells were harvested and surface-stained with anti-CD3 mAb APC (BioLegend, San Diego, California, USA; cat. 344812), anti-CD4 mAb APC-Cy7 (BioLegend, San Diego, California, USA; cat. 317418), and anti-CD8 mAb FITC (BioLegend, San Diego, California, USA; cat. 344704) for PBMCs from critically ill patients hospitalized during the first wave of COVID-19. For PBMCs from the other study groups, cells were stained with anti-CD3 mAb AF647 (BioLegend, San Diego, California, USA; cat. 300322), anti-CD4 mAb APC/Cy7 (BioLegend, San Diego, California, USA; cat. 317418), and anti-CD8 mAb PerCP/Cy5.5 (BioLegend, San Diego, California, USA; cat. 300924). The staining mixture was incubated for 15 minutes at room-temperature in the dark, followed by an additional washing step with cell staining buffer. Subsequently, the cells were fixed and permeabilized using Cytofix/Cytoperm for 20 minutes at room-temperature. After centrifugation, Perm/Wash was added. Intracellular staining was then performed using anti-interferon (IFN)-γ mAb PE (BioLegend, San Diego, California, USA; cat. 506507) for critically ill patients hospitalized and anti-IFN-γ mAb FITC (BioLegend, San Diego, California, USA; cat. 502506) for PBMCs from the other study groups in Perm/Wash solution (BD, San Jose, USA) for 30 minutes at room-temperature in the dark. After washing with BD Perm/Wash buffer (BD, San Jose, USA), the cells were resuspended in PBS and kept at 4°C in the dark until acquisition and analysis. Flow cytometric analysis was performed using the FACS Canto II flow cytometer (BD Biosciences, New Jersey, USA), and the data were analyzed using FlowJo V10 software (**Supplementary Figure 1**).

### Statistical analysis

Descriptive statistics were used to analyze the variables of interest. For discrete or continuous quantitative variables, the mean, median, and standard deviation were determined as appropriate for each case. The choice between parametric or non-parametric statistics was based on the distribution and variance of the data, following the Kolmogorov-Smirnov normality test. Antibody titers were analyzed using Student’s t-test or the Mann-Whitney U test for two categories, while cell percentages were analyzed using the Kruskal-Wallis and Dunn’s multiple comparisons test. The data were processed using Excel, the GraphPad Prism analysis program, and STATA v.14 software.

## Results

### Subject characteristics

A total of 56 subjects were enrolled for the present study. Out of these, 28 (50%) are male and 28 (50%) are female. Within the analyzed population, we observed a predominance of type II diabetes mellitus and obesity; however, we did not find significant differences between the analyzed groups (**Table 1**).

**Table 1:** Demographic characteristics.

n (%)	COVID-19 Acu 12 (21)	COVID-19 Acu Rec 6 (11)	COVID-19 VOC 10 (18)	Vac mRNA 15 (27)	Vac mRNA+AZ 13 (23)
**Age, mean (SD)**	59.8 (15.5)	54.5 (18.9)	52.67 (23.4)	43.8 (14.2)	40.9 (7.5)
Gender n (%)					
**Male**	9 (75)	5 (83)	5 (50)	5 (33)	4 (31)
**Female**	3 (25)	1 (17)	5 (50)	10 (67)	9 (69)
Comorbidities n (%)					
**Diabetes Mellitus 2**	5 (42)	2 (33)	1 (10)	0	0
**Hypertension**	4 (33)	1 (17)	2 (20)	1 (7)	1 (8)
**Obesity**	2 (17)	1 (17)	4 (40)	1 (7)	1 (8)

Acu, acute phase; Rec, Recovered patients; VOC, Variants of Concern; Vac mRNA, Vaccinated with BNT162b2; Vac mRNA+AZ, Vaccinated with BNT162b2 and AZ/ChAdOx-1-S; SD, Standard Deviation.

### Analysis of antibody response for antigenicity evaluation of the AVX/COVID-12 vaccine

**Figure 1:**
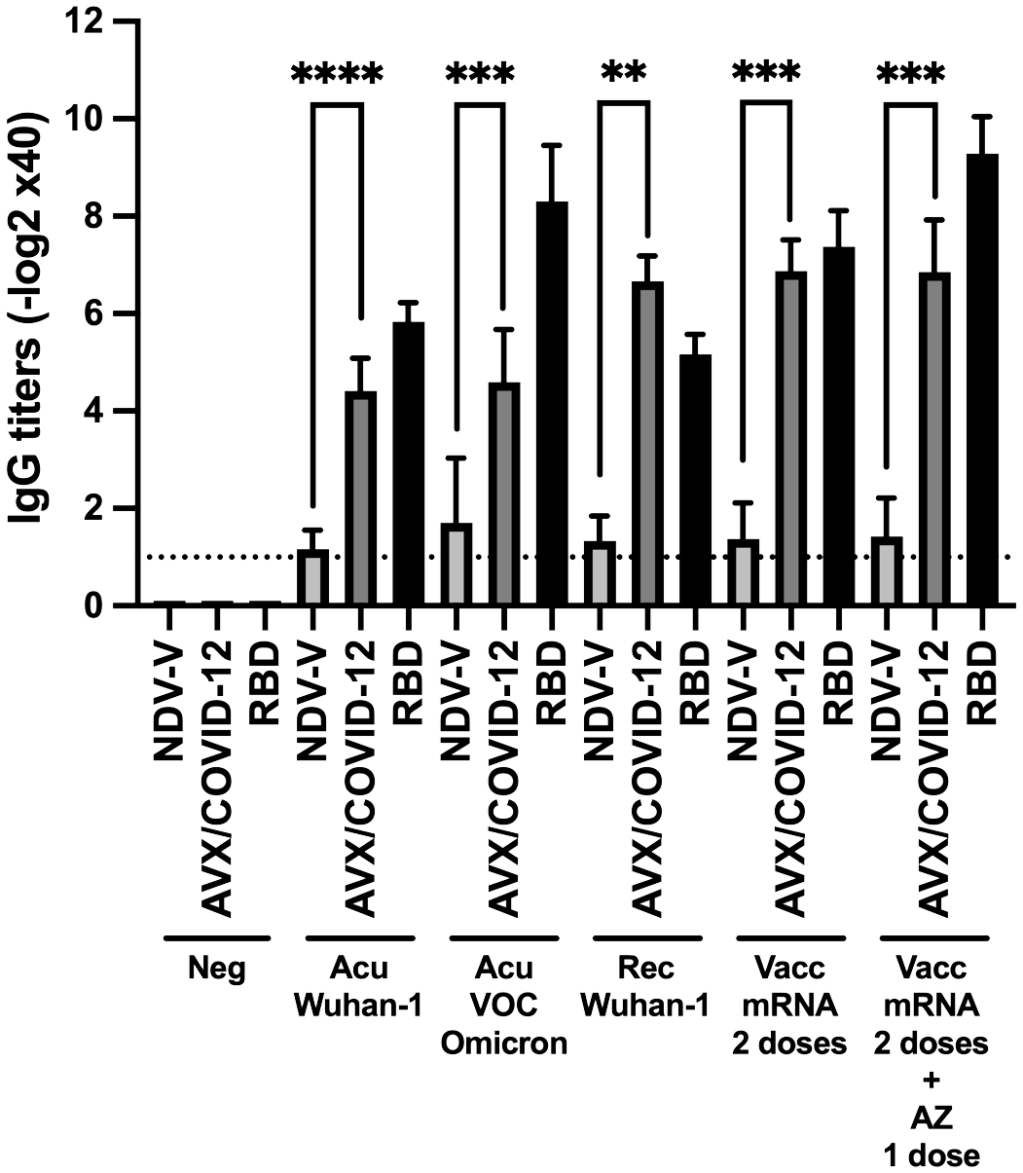
Binding of serum IgG from COVID-19 patients and vaccinated individuals to the AVX/COVID-12 vaccine and the Receptor-Binding Domain (RBD) of the spike protein of SARS-CoV-2. ELISAs were conducted to assess the binding of the AVX/COVID-12 vaccine and the receptor binding domain (RBD) to serum IgG antibodies from patients in the acute phase of COVID-19 (Acu Wuhan-1; n=12) and a subgroup with the Omicron variant (Acu VOC Omicron; n=10), as well as individuals who had recovered from the disease (Rec Wuhan-1; n=6). Additionally, samples were collected from individuals vaccinated with two doses of the BNT162b2 Pfizer vaccine after 6 months of the second dose (Vacc mRNA; n=15) and those who received two doses of BNT162b2 and were boosted with the AZ (AZ/ChAdOx-1-S; n=13) vaccine after 3 months of the third dose. Control groups included NDV-V (NDV LaSota virus empty vector), AVX/COVID-12 (NDV expressing SARS-CoV-2 S protein and RBD) and Neg (pre-pandemic sera n=6). Mann-Whitney two-tailed unpaired test was used, with significance set at p < 0.05. The detection limit is represented by the dashed line, which corresponds to the initial serum dilution tested at 1:40. p < 0.01**, p < 0.001*** and p < 0.0001****.

To evaluate the antigenicity of AVX/COVID-12, we performed *in vitro* tests with antibodies induced after infection or vaccination against COVID-19. Utilizing ELISA, we assessed the binding of these antibodies to the AVX/COVID-12 vaccine, NDV-V, and recombinant RBD (**Figure 1**).

We observed specific AVX/COVID-12 IgG antibody titers ranging from 4 to 7 logarithmic units base 2 (log2 units) in sera from both acute and recovered SARS-CoV-2 infected patients. Notably, sera from hospitalized patients during the Omicron wave, who had not received prior COVID-19 vaccination in Mexico, also exhibited binding to AVX/COVID-12, similar to sera from patients infected with other VOCs. In vaccinated individuals, we observed statistically significant differences with higher titers, ranging from 7 to 9. In contrast, NDV-V, which lacks SARS-CoV-2 spike protein expression, exhibited only marginal binding with a titer of 1. Using RBD as a positive control for coating the plates, we found no statistical differences between antibody titers binding to RBD or the AVX/COVID-12 vaccine. These results suggest that the spike protein expressed in the AVX/COVID-12 vaccine enables the binding of IgG antibodies triggered by the spike protein expressed by the SARS-CoV-2 variant that infected patients, as well as by the BNT162b2 and AZ/ChAdOx-1-S vaccines.

### AVX/COVID-12 vaccine elicits T-cell proliferation and IFN-γ production in patients and COVID-19 vaccinated volunteers

**Figure 2:**
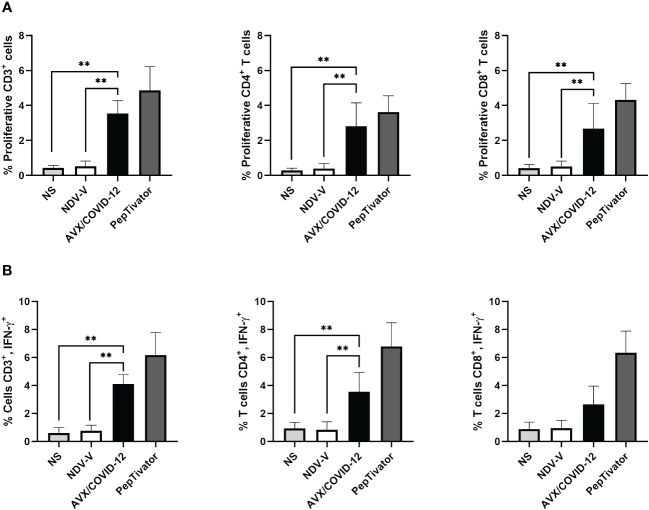
AVX/COVID-12 vaccine induces proliferation and IFN-γ response in T-cells from acute COVID-19 patients. Proliferation response **(A)** or the percentage of IFN-γ+ **(B)** in T cells from acute COVID-19 patients (ancestral first wave) was assessed following stimulation with NDV-V (empty vector of NDV LaSota virus), AVX/COVID-12, or PepTivator (S1 and S2 peptides) for 72 hours. Flow cytometry analysis encompassed individual evaluations of CD4+ and CD8+ responses in single, FSClow SSClow CD3+ cells. NS (Not Stimulated). n=6, Kruskal-Wallis test followed by Dunn’s multiple comparisons test. The asterisks (*) indicate significant differences. Adjusted p-values were used to correct for multiple comparisons: p < 0.01**.

**Figure 3:**
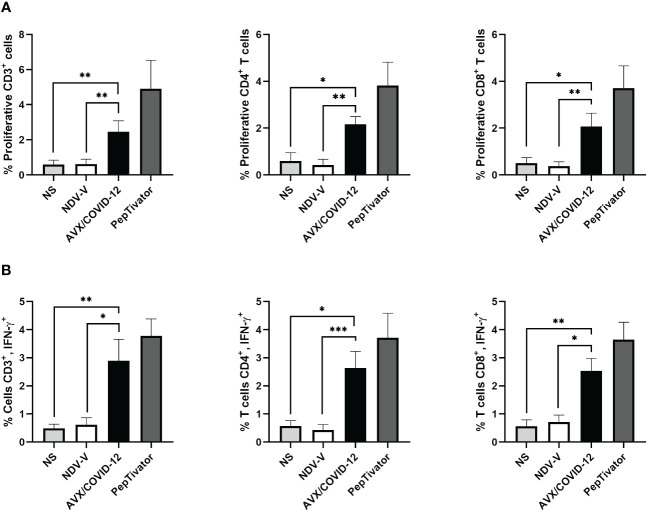
AVX/COVID-12 vaccine elicits proliferation and IFN-γ response in T-cells from individuals vaccinated with two doses of mRNA BNT162b2. Proliferation response **(A)** or percentage of IFN-γ+ **(B)** in T cells stimulated with NDV-V (empty vector of NDV LaSota virus), AVX/COVID-19 vaccine, or PepTivator (S1 and S2 peptides) for 72 hours. Flow cytometry analysis includes single, FSClow SSClow CD3+ cells, and individual responses of CD4+ and CD8+, respectively. NS (Not Stimulated). n=10, Kruskal-Wallis test followed by Dunn’s multiple comparisons test. The asterisks (*) indicate significant differences. Adjusted p-values were used to correct for multiple comparisons: p < 0.05*, p < 0.01** and p < 0.001***.

Next, we investigated whether the AVX/COVID-12 vaccine activates T-cells in individuals who had COVID-19 or were vaccinated with anti-COVID-19 vaccines. We stimulated PBMCs from patients (**Figure 2**) and vaccinated volunteers (**Figure 3**) with AVX/COVID-12 or the NDV-V empty vector. As a positive control, we utilized a spike protein peptide cocktail mixture of 10–15 amino acid peptides (PepTivator S) (**Supplementary Figure 1**).

We observed that the AVX/COVID-12 vaccine induced specific proliferation and intracellular IFN-γ production in both critical COVID-19 patients and vaccinated volunteers. In contrast, NDV-V induced a basal response similar to that of non-stimulated cells (**Figures 2A**, **3A**). The positive control, PepTivator, also triggered strong specific cell proliferation and IFN-γ intracellular production, surpassing AVX/COVID-12 vaccine by 1–2%.

**Figure 4:**
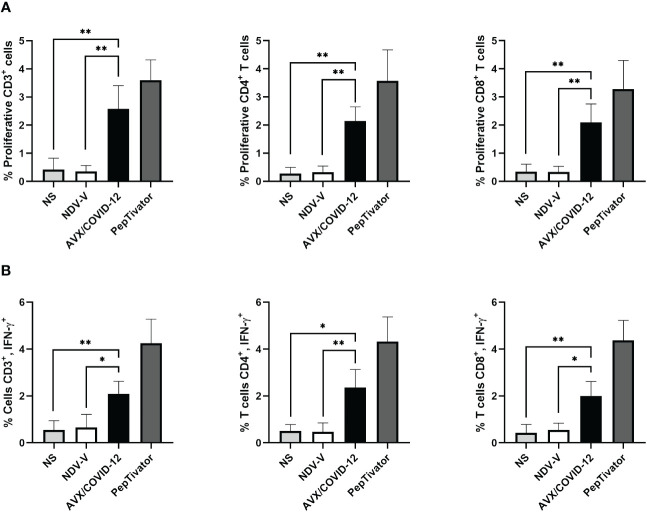
AVX/COVID-12 vaccine induces proliferation and IFN-γ response in T-cells from individuals vaccinated with two doses of mRNA BNT162b2 and boosted with AZ/ChAdOx-1-S vaccine. Proliferation response **(A)** or percentage of IFN-γ+ **(B)** in T cells stimulated with NDV-V (empty vector of NDV LaSota virus), AVX/COVID-12, or PepTivator (S1 and S2 peptides) for 72 hours. Flow cytometry analysis includes single, FSClow SSClow CD3+ cells and individual response of CD4+ and CD8+ respectively. NS (no stimulated). n=10, Kruskal-Wallis test followed by Dunn’s multiple comparisons test. The asterisks (*) indicate significant differences. Adjusted p-values were used to correct for multiple comparisons: p < 0.05*, p < 0.01**.

The production of intracellular IFN-γ in total CD3+ T cells from patients (**Figure 2B**) was higher than that observed in volunteers vaccinated with two (**Figure 3B**) and three doses (**Figure 4B**) (p = 0.0022, p = 0.0016 and p = 0.0073). The same trend was observed for CD4+ T-cells (p = 0.0087, p = 0.0114 and p = 0.0035) and CD8+ T-cells (p = 0.0649, p = 0.0016 and p = 0.0035). Additionally, we observed an increase in the proliferation response in T-cells from critically ill acute patients compared to those vaccinated with two doses of the BNT162b2 vaccine (**Figures 2A**, **3A**).

With a vaccine booster, one would anticipate a substantial increase in both antibody and cellular responses, potentially ranging from 10 to 25 times greater (16, 17). Therefore, we examined the cellular response in PBMCs from volunteers who had received two doses of BNT162b2 and were subsequently boosted with the AZ/ChAdOx-1-S vaccine, stimulated with the AVX/COVID-12 vaccine (**Figure 4**). We detected specific proliferation and intracellular production of IFN-γ induced by the AVX/COVID-12 vaccine in CD3+, CD4+, and CD8+ T-cells, while the NDV-V empty vector induced basal activation similar to that of non-stimulated cells. Nevertheless, the response in volunteers who received a booster dose with the AZ/ChAdOx-1-S vaccine is comparable to that of volunteers who received only two doses of the BNT162b2 vaccine, with a slight tendency to be lower when compared to the response observed in T-cells from critically ill COVID-19 patients.

## Discussion

During the SARS-CoV-2 pandemic, there was a pressing need for accelerated technological advancements in the development of new vaccine platforms. mRNA vaccines and viral vectors emerged as frontrunners, receiving emergency use approvals, and demonstrating their efficacy in alleviating hospital saturation and reducing deaths due to COVID-19. However, to achieve broader vaccination coverage, especially in LMICs, there is a demand for vaccine candidates that are not only effective but also affordable and easily transportable. The NDV viral vector platform has proven to be a safe and innocuous vector (9, 10, 18). It is easily developed for use, showing high effectiveness in oncolytic treatment and preclinical models as a live vaccine vector (19, 20). This makes it a promising candidate for meeting vaccine needs in diverse settings. Another advantage is that it can be easily and inexpensively produced in manufacturing facilities similar to those used for influenza vaccine. Furthermore, this active vaccine could be delivered intranasally, which would enhance its acceptance among the population. A pilot and good manufacturing practice (GMP) production facility has been established in Mexico to address the lack of infrastructure for vaccine development and manufacturing in the region.

The use of the NDV-HXP-S (AVX/COVID-12-HEXAPRO) vector technology has facilitated the development of the AVX/COVID-12 vaccine candidate, demonstrating effectiveness in eliciting neutralizing antibodies and an IFN-γ^+^ T-cell response crucial for combating SARS-CoV-2 infection (9, 11).

Due to the updated COVID-19 vaccine distribution inequities affecting mainly LMICs, it is crucial to gather evidence on the efficacy of currently available vaccines and vaccine candidates. This should focus on assessing the capacity of the spike protein from the original Wuhan-1 strain to generate immunity against emerging VOCs such as Omicron and its subvariants.

If the epitopes constituting the spike protein of the SARS-CoV-2 or those from the first-generation vaccines (BNT162b2 and AZ/ChAdOx-1-S) are efficiently expressed in the NDV vector, we anticipate the development of antibodies targeting similar epitopes. These antibodies would play a crucial role in protecting the host from the development of severe and critical pathology (21–23).

One approach to evaluate the capacity of a vaccine candidate in eliciting immune responses in humans involves *ex vivo* tests assessing its antigenicity. This process entails examining the candidate’s interaction with pre-existing antibodies developed in patients who have recovered from the targeted pathology or through the recognition and activation of the cellular immune response.

We observed the binding of antibodies to the spike protein in comparison to the empty vector NDV-V or the RBD in different study groups during and after infection or post-vaccination with second and third doses. Anti-spike IgG titers showed an increase of 4 to 6 log2 units across all study groups (**Figure 1**), indicating similar humoral responses between the two vaccination schemes. In this context, the design of the vaccine protein S in BNT162b2 and AVX/COVID-12 shares the prefusion-stabilized structure (10). However, there are differences in the substitutions made to maintain the structure of the molecule, involving two prolines in BNT162b2 and six prolines in the S2 subunit of AVX/COVID-12 (10, 24). This suggests antibodies are targeting conformational epitopes allowing both candidates to potentially share structural epitopes. When comparing the antigenicity against antibodies developed with a third dose of the AZ/ChAdOx-1-S vaccine, where the structure of the protein S resembles the native one (25), we observed similar activation (without significant differences, p > 0.9) compared to the complete scheme with BNT162b2 alone. Therefore, all three designs appear to induce similar repertoires of antibodies against SARS-CoV-2.

Moreover, upon analyzing the antibody response in patients during the acute phase and those who have recovered from COVID-19, we observed that recovered patients had 3 log2 units higher anti-spike IgG antibody titers than acute patients (**Figure 1**). In contrast, it has previously been reported that there are no significant differences in antibody titers among acute and recovered patients (26). However, differences in the quality of the response are observed, particularly in terms of opsonization, complement activity, and neutralizing capability (27). This is attributed to the significant reduction in the percentage and total numbers of T and B lymphocyte populations during severe acute COVID-19. This reduction reflects a noteworthy decrease in the development of germinal centers in lymph nodes, potentially impairing the affinity and repertoire of antibodies to various epitopes of the total spike protein (28, 29). The increase of antibody titers in recovered patients suggests a gradual increase in the number of epitopes recognized by the immune system during the recovery phase from COVID-19.

In the design of vaccines or platforms, the evaluation of T-cell activation response has been a key aspect. For instance, in cases like the respiratory syncytial virus in infants or the pandemic influenza H1N1 virus, assays involving lymphoproliferation and IFN-γ secretion have been utilized as indicators of T-cell effector activity, providing valuable insights into the vaccine’s effectiveness (30, 31). We analyzed three pivotal scenarios during the pandemic: the cellular response in COVID-19 patients, emphasizing the effector response; the vaccination schemes, specifically 6 months after the second dose to ensure memory T-cell stimulation in peripheral blood; and 3 months after the third dose, exemplifying restimulation with a heterologous immunization schedule. The analysis revealed that, 72 h after stimulation, proliferation was induced with the vaccine in the case of patients (**Figures 3**, **4**). This was expected, as despite a low percentage of T-cells in patients, they exhibited a robust effector response primarily targeting spike and nucleocapsid protein epitopes. Consequently, these T-cells were activated by AVX/COVID-12 and produced IFN-γ. Regarding vaccinated volunteers, notable differences were observed in both cases. Surprisingly, when comparing proliferation and interferon responses, they appeared to be similar between both doses (data not reported). This contradicts expectations, since with a vaccine booster, one would anticipate a substantial increase in both antibody and cellular responses, ranging from 10 to 25 times greater (16, 17). However, since we are examining antigenicity, we can only illustrate the epitopes present in the vaccine. Therefore, upon immunization with the candidate, we anticipate the response to escalate. These observations are consistent with the identification of a substantial number of conserved epitopes within the spike protein, contributing to both humoral and cellular responses across ancestral SARS-CoV-2 and its variants of concern, as well as SARS-CoV, MERS-CoV, and seasonal coronaviruses (32, 33). This may even extend to other coronaviruses within the *Orthocoronavirinae* genus (34).

In conclusion, the AVX/COVID-12 vectored vaccine candidate demonstrates the ability to stimulate robust cellular responses and is recognized by antibodies primed by the spike protein present in SARS-CoV-2 viruses that infected patients, as well as in the mRNA BNT162b2 and AZ/ChAdOx-1-S vaccines. These findings endorse the integration of the AVX/COVID-12 vaccine as a booster in vaccination initiatives designed to combat COVID-19 caused by SARS-CoV-2 and its VOCs.

### Study limitations

We cannot rule out the possibility of VOCs patients who did not report or were asymptomatic for previous SARS-CoV-2 infections, which could affect our ability to test the capacity of the AVX/COVID-12 vaccine to stimulate their T cells.

## Data availability statement

The raw data supporting the conclusions of this article will be made available by the authors upon request to any researchers with a sound analytical proposal, without undue reservation.

## Ethics statement

The protocol was approved by the ethics, research, and biosafety committees of the Mexican Institute of Social Security (IMSS) National Scientific Research and Ethics Commissions. Informed consent was obtained from both patients and vaccinated individuals. This project was conducted in accordance with good clinical practice. Project authorization numbers R-2020-785-095 and R-2021-785-048.

## Author contributions

AT-F: Data curation, Formal Analysis, Investigation, Visualization, Writing – original draft, Writing – review & editing. LO-P: Formal Analysis, Investigation, Writing – review & editing. RM-S: Data curation, Formal Analysis, Investigation, Writing – review & editing, Methodology. AT-S: Data curation, Writing – review & editing. JG-M: Data curation, Writing – review & editing. TR-H: Data curation, Formal Analysis, Investigation, Supervision, Validation, Visualization, Writing – review & editing. EF-O: Data curation, Formal Analysis, Writing – review & editing. AC-V: Data curation, Formal Analysis, Writing – review & editing. LA-P: Data curation, Formal Analysis, Methodology, Validation, Writing – review & editing. LB: Data curation, Formal Analysis, Methodology, Validation, Writing – review & editing. GP-D: Methodology, Writing – review & editing. OR-M: Investigation, Methodology, Resources, Writing – review & editing. A-SM: Methodology, Resources, Writing – review & editing. GP-S: Project administration, Writing – review & editing. DS-M: Funding acquisition, Resources, Writing – review & editing. WS: Formal Analysis, Resources, Writing – review & editing. HC-C: Conceptualization, Formal Analysis, Funding acquisition, Project administration, Supervision, Writing – review & editing. PP: Writing – review & editing, Formal Analysis, Resources. FK: Formal Analysis, Resources, Writing – review & editing. AG-S: Formal Analysis, Resources, Writing – review & editing. BL-D: Resources, Writing – review & editing, Conceptualization, Funding acquisition, Supervision, Validation. CL-M: Conceptualization, Funding acquisition, Resources, Supervision, Validation, Writing – review & editing, Data curation, Formal Analysis, Investigation, Project administration, Visualization, Writing – original draft.
