# Supplementary material for: Newcastle disease virus vector-based SARS-CoV-2 vaccine candidate AVX/COVID-12 activates T cells and is recognized by antibodies from COVID-19 patients and vaccinated individuals
Source: Front Immunol. 2024 May 30;15:1394114. doi: 10.3389/fimmu.2024.1394114 (PMC11169921; doi:10.3389/fimmu.2024.1394114)
Supplement: Supplementary file 1 [file DataSheet_1.pdf]

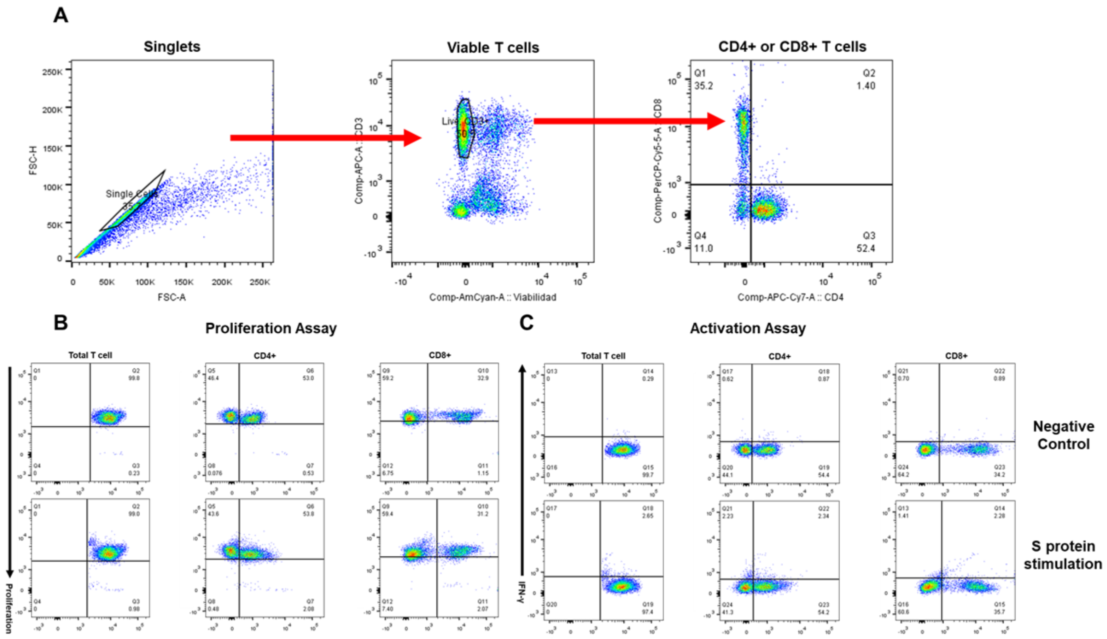

Figure 1S. Flow cytometry gating strategy. Representative PBMCs sample gating hierarchy. From left to right and top to bottom: (A) FSC-A versus SSC-A for cellular viability and identification of $CD4^{+}$ or $CD8^{+}$ T cells, (B) proliferation assay, and (C) individual T cell responses to produce IFN-γ.
